# Characteristics and Prognostic Factors Associated With the Progression of Myopic Traction Maculopathy in Mexican Patients

**DOI:** 10.7759/cureus.64036

**Published:** 2024-07-07

**Authors:** Mauricio Bayram-Suverza, Carla Pérez-Montaño, Jose Rafael Villafán-Bernal, Sergio Rojas-Juárez, Arthur Levine-Berebichez, Juan Abel Ramírez-Estudillo

**Affiliations:** 1 Retina, Fundación Hospital Nuestra Señora de la Luz, Mexico, MEX; 2 Immunogenomics and Metabolic Diseases, Instituto Nacional de Medicina Genómica, Mexico, MEX

**Keywords:** optical coherence tomography, visual acuity, myopia progression, myopic traction maculopathy, high myopia

## Abstract

Background

In this study, the characteristics and prognostic factors associated with the progression of myopic traction maculopathy (MTM) were evaluated in a Mexican population.

Methods

This is a retrospective observational study that analyzed patients with MTM who underwent optical coherence tomography (OCT). Clinical-ocular information, the MTM classification, and initial and final visual acuity (VA) were recorded.

Results

In total, 101 eyes of 84 patients (mean age 63.5 ± 10.7 years) were included (88.1% female and 11.9% male). The mean spherical equivalent was -16.8 ± 6.4 D, axial length was 29.6 ± 2.1 mm, and mean initial VA was 0.8 ± 0.5 logMAR. The mean follow-up time was 25.7 ± 27.6 months. The change in final VA from diagnosis to the last follow-up was +0.1 (0.2) (p = 0.001). Overall, 24.8% of patients progressed, 72.3% did not progress, and 3% showed regression. The patient-year progression rate was 0.20 ± 0.44. Factors associated with progression were initial logMAR VA (p= 0.012) and staphyloma (p= 0.001).

Conclusions

One in four patients with MTM progressed, and the patient-year progression rate was 0.5. The factors associated with disease progression were initial VA and the presence of staphyloma. The characteristics of Mexican patients with MTM are similar to those described in other populations.

## Introduction

Myopia is a common eye condition with an increasing prevalence worldwide, particularly in Asian countries. Associated macular complications can result in visual impairment and blindness [1]. In 2020, myopia-related macular complications accounted for 17% of global visual impairment cases. If the current epidemiological trend persists, this percentage is expected to increase by 58% by 2050 [2,3].

The Los Angeles Latino Eye Study found that pathological myopia is the third leading cause of blindness among Mexicans and their descendants. The study revealed that Latinxs have a high frequency (12.5%) of blindness related to pathological myopia [4].

High myopia is characterized by a refractive error greater than six diopters (D) or an axial length (AL) greater than 26.0 mm. When associated with degenerative changes in the posterior pole, it becomes pathologic myopia [5,6]. Compared with lesser degrees of myopia, high myopia often progresses to chorioretinopathies, including chorioretinal atrophy, tractional lesions, and choroidal neovascularization [7].

Myopic tractional maculopathy (MTM) is a group of pathological changes detected in the macular areas of patients with high myopia on optical coherence tomography (OCT). It includes a range of clinical conditions, such as vitreomacular traction, epiretinal membrane, maculoschisis, foveal detachment, and internal, external, and full-thickness lamellar macular holes [8,9].

Maculoschisis is a common lesion observed in patients diagnosed with MTM. It has a high incidence rate among people with high myopia and posterior staphyloma, affecting approximately 31.3% of these patients. Maculoschisis can present either foveally or extrafoveally and occur at different levels between the layers of the neurosensory retina [10].

Parolini et al. recently developed the MTM staging system (MSS), which characterizes retinal changes caused by the anteroposterior and tangential forces involved in the evolution of MTM [9]. MTM has a wide clinical spectrum, and variation among populations has not been fully determined. This study aimed to identify the characteristics and risk factors associated with the progression of MTM in a Mexican population through the assessment of macular OCT. The results provide insight into the specific features of MTM in Mexican patients and provide a basis for comparisons with those observed in other populations.

## Materials and methods

Ethics statements

This study adhered to the guidelines of the Helsinki Declaration and had the approval of the local Ethics Committee of the hospital “Fundación Hospital Nuestra Señora de la Luz” and the Institutional Review Board. Approval code: 2023R21B2. All patients signed written informed consent for participation.

Study design and population

In this retrospective study, data of interest were extracted from the medical records of all patients with high myopia who visited the Retina Department of Nuestra Señora de Luz Hospital in Mexico City, Mexico, between January 2014 and December 2022. For study inclusion, patients had to be followed up for at least six months and OCT measures. The exclusion criteria were poor-quality OCT images, eyes with a history of vitreoretinal surgery, and eyes with ocular diseases other than MTM that can cause foveoschisis.

Data collection

The diagnosis of MTM was based on the MSS reported by Parolini et al [9]. High myopia was defined as a spherical equivalent (SE) refractive error of ≥ 6 D or an AL of ≥ 26 mm [9].

Patient ethnicity was identified through a review of medical records. Data regarding age (years), sex (male/female), eye (right/left), and Snellen visual acuity (best corrected visual acuity (BCVA)) were collected and converted to logMAR for analysis. AL measurements were performed when available. All available images were retrieved from the Spectralis OCT database (Heidelberg Engineering, Heidelberg, Germany).

According to the MSS, all OCT studies were classified as follows. Four stages (reported in four rows on the vertical axis, showing the evolution of the retinal layers perpendicular to the plane of the retina) were defined as follows: stage 1, inner or inner-outer maculoschisis; stage 2, predominantly outer maculoschisis; stage 3, association between maculoschisis and retinal detachment; and stage 4, retinal detachment [9].

Foveal changes (reported in three columns on the horizontal axis to emphasize the evolution of the retinal layers in a tangential orientation to the retinal plane) were defined as follows: A, absence of splitting in the fovea; B, presence of a lamellar macular hole in the fovea; and C, presence of a full-thickness macular hole in the fovea. External macular holes and epiretinal membranes were reported separately by adding "+" and "0,” respectively.

Progression and regression, defined as the worsening and improvement of categories, respectively, were documented. The eyes that did not meet the definitions of progression or resolution were classified as stable.

The presence and classification of MTM on SD-OCT images were assessed by two graders (MB and CP). The opinion of a third specialist (JAR) was asked in cases of discordance.

Statistical analysis

Statistical analyses were performed using Statistical Product and Service Solutions (SPSS, version 26; IBM SPSS Statistics for Windows, Armonk, NY). The distribution of quantitative variables was estimated by the Kolmogorov-Smirnov test. If the distribution was normal, then means and standard deviations were employed otherwise median and interquartile range was reported. Qualitative data were estimated through frequencies and percentages. The paired t-test was used to detect statistically significant differences in logMAR values after follow-up. The regression and progression rates by patient-year were calculated as the proportion of total patients that progressed or regressed by year. A binary logistic regression analysis with the Enter method was used to identify factors related to the progression of MTM, obtaining the odds ratio (OR) (95% confidence interval (CI)) and β-coefficient. Statistical significance was set at p < 0.05.

## Results

Demographic characteristics

This study included 101 eyes of 84 patients with MTM (88.1% female and 11.9% male). Of the affected eyes, 49.5% were the left eye (n = 50) and 50.5% were the right eye (n = 51). The mean age of the patients was 63.5 ± 10.7 years (range: 36-93 years).

Clinical characteristics

The mean SE of patients with MTM was -16.8 ± 6.4 D, AL was 29.6 ± 2.1 mm, and mean logMAR VA was 0.8 ± 0.5 (Table 1). The initial classifications of MTM are presented in Table 2. The most common type was type 2A, followed by 2B (Table 2).

**Table 1:** Ocular characteristics of patients with myopic tractional maculopathy. D: diopter; BCVA: best corrected visual acuity

Characteristic	Value
Spherical equivalent (D)	-16.8±6.4
Axial length (mm)	29.6±2.1
BCVA (logMAR)	0.8±0.5
Pseudophakia	25.7 (26)
Neovascular membrane	12.9 (13)

**Table 2:** Basal MTM classification of patients (n=101). MTM: myopic traction maculopathy

Stage	n (%)
0	5.9 (6)
1A	5.9 (6)
1A+	3.0 (3)
1B	5.0 (5)
1C	1.0 (1)
2A	33.7 (34)
2A+	4.0 (4)
2B	17.8 (18)
2B+	3.0 (3)
3A	2.0 (2)
3B	3.0 (3)
3B+	1.0 (1)
3C	1.0 (1)
4A	5.9 (6)
4A+	1.0 (1)
4AO	1.0 (1)
4C	5.9 (6)

Follow-up

The mean follow-up duration of patients with MTM was 25.7 ± 27.6 months (range, 6-108 months), and the mean number of follow-up visits was 3.7 ± 2.8. Of the patients, 43.6% had two follow-up visits, 28.7% had three follow-up visits, and the remaining 28.7% had four or more follow-up visits.

Changes in the BCVA and MTM stage during the follow-up

**Figure 1:**
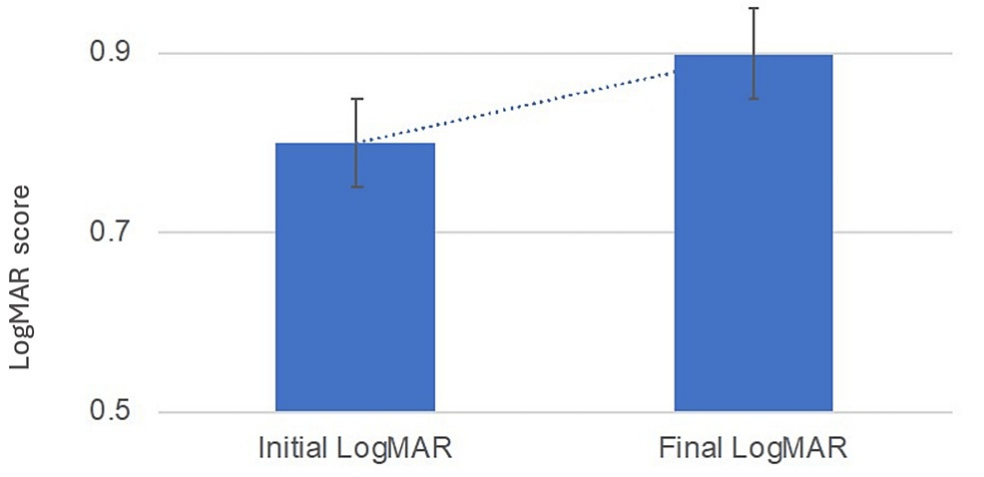
Visual acuity changes between diagnosis and the last follow-up (p < 0.001, paired t-test). Note: the top of the bar represents the mean, and the whisker represents the standard deviation.

logMAR VA at the end of the follow-up was 0.9 ± 0.5, whereas it was 0.8 ± 0.5 at the beginning of the study. Thus, there was an increase in logMAR of +0.1 (0.2) (p < 0.001, paired t-test; Figure 1).

The MSS classifications at the end of the follow-up are presented in Table 2. The most frequent categories were 2A (34.7%), 2B (15.8%), and 4A (9.9%) (Table 3).

**Table 3:** Classification of patients with MTM at the final follow-up. MTM: myopic traction maculopathy

MTM Classification	N (%)
0	2.0 (2)
1A	2.0 (2)
1A+	2.0 (2)
1B	4.0 (4)
1C	1.0 (1)
2A	34.7 (35)
2A+	5.9 (6)
2B	15.8 (16)
2B+	3.0 (3)
3A	5.0 (5)
3AO	1.0 (1)
3B	3.0 (3)
3B+	1.0(1)
4A	9.9 (10)
4A+	1.0 (1)
4AO	1.0 (1)
4B+O	1.0 (1)
4C	6.9 (7)

Natural course of MTM

**Figure 2:**
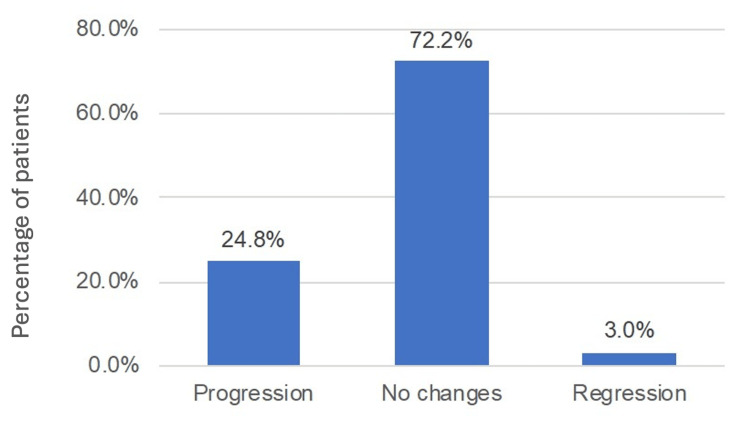
Disease progression and regression in patients with myopic traction maculopathy.

**Figure 3:**
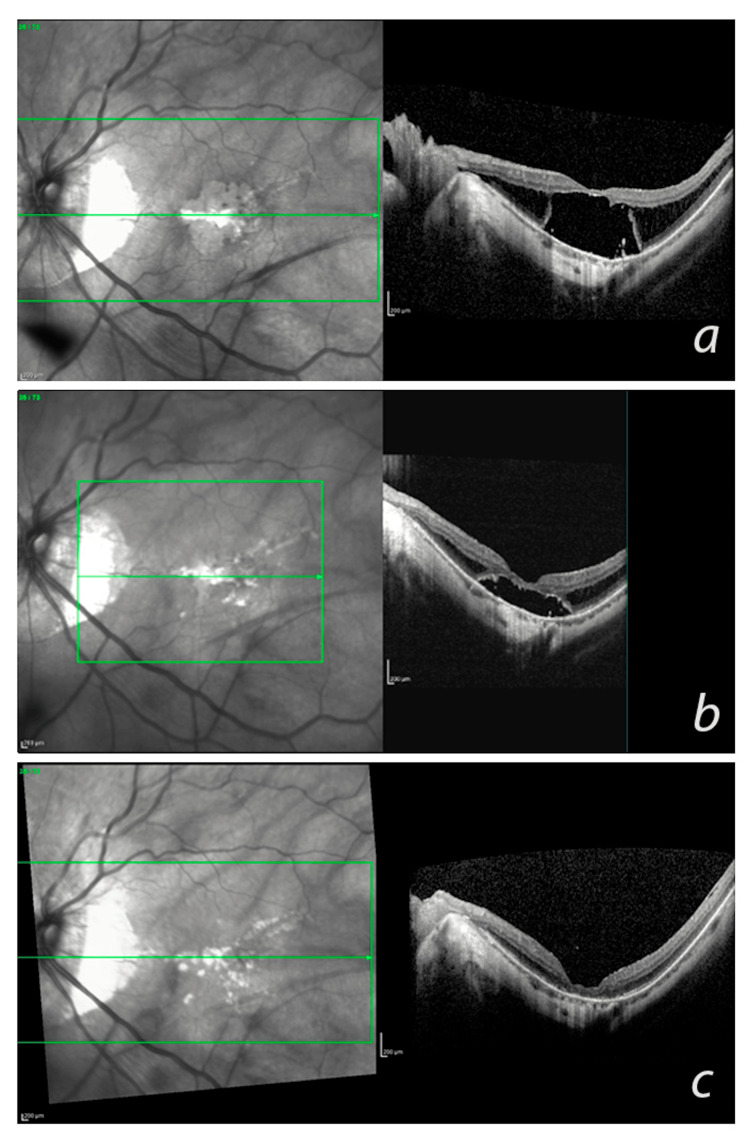
Representative case of myopic traction maculopathy (MTM) regression. (a) During the basal examination of the patient, a macular optical coherence tomography showed macular detachment at stage 3A. (b) At the 12th month of follow-up, the macular detachment progressively collapsed. (c) After 30 months, the retina was reattached, and MTM was resolved; however, there was predominant macular atrophy of the external layers.

**Figure 4:**
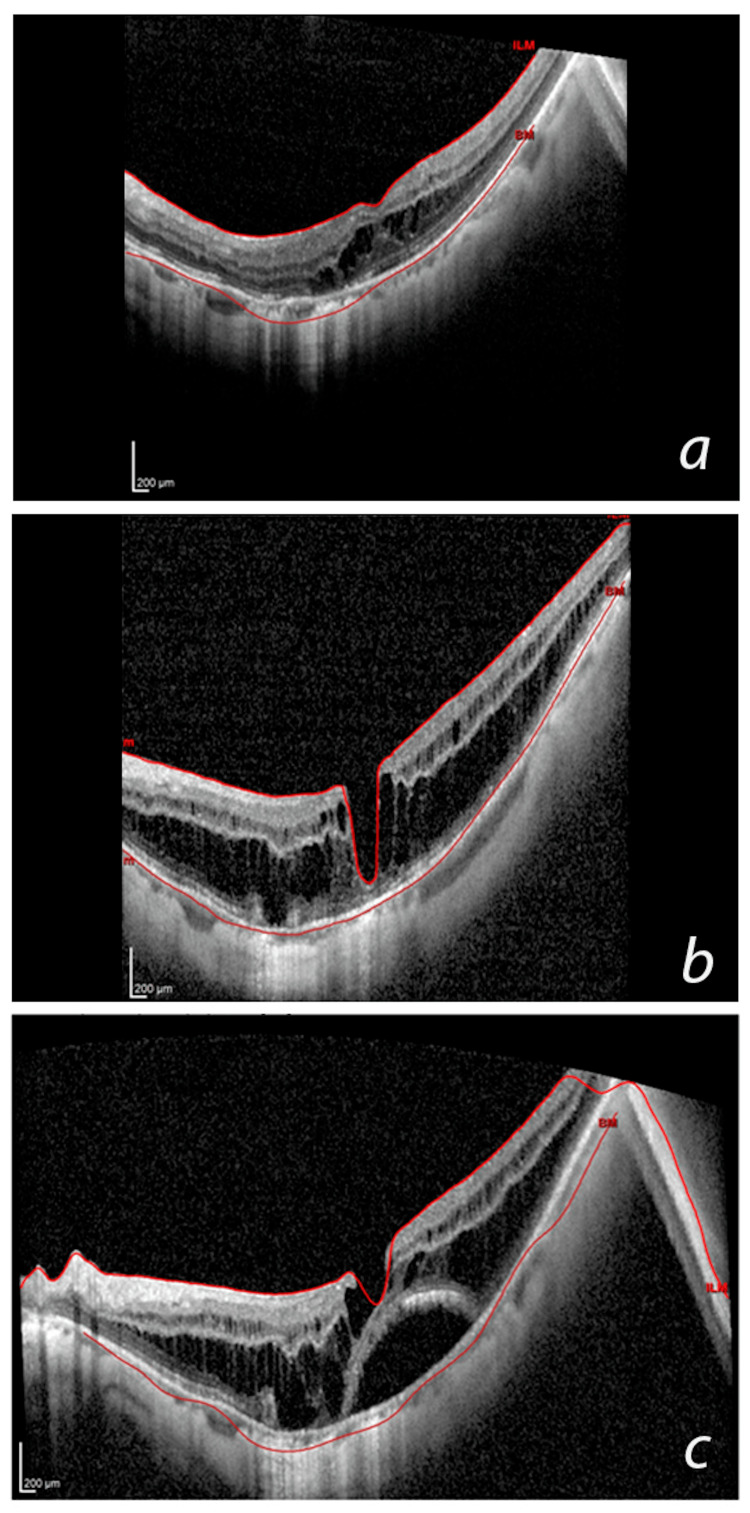
Representative case of myopic traction maculopathy (MTM) progression. During the initial OCT examination (a), maculoschisis was found predominantly in the outer nuclear layer (stage 2A). After 36 months (b), the patient presented with maculoschisis in both the inner and outer retinal layers (stage 2A). After 42 months (c), the patient developed a lamellar macular hole and macular detachment, accompanied by the previous findings (stage 3B).

Of the patients with MTM, 24.8% progressed (Figure 2). The regression rate was 0.007 patients/year (Figure 3). The patient-year progression rate was estimated as 0.20 ± 0.44 patients/year (Figure 4).

Factors associated with the progression of MTM

To identify the factors associated with the progression of MTM, a binary logistic regression analysis was performed using the “enter” method, identifying initial logMAR VA (β = -1.501, OR: 0.223, 95% CI: 0.069-0.719, p = 0.012) and the presence of staphyloma as significant factors (β = 2.035, OR: 7.650, 95% CI: 2.225-25.947, p = 0.001) (Table 4). That is, the lower the LogMAR (better VA) value, the lower the probability of progression.

**Table 4:** Factors associated with the progression of myopic traction maculopathy. BCVA: best corrected visual acuity, OR: odds ratio, CI: confidence interval, Inf: infinity

Factor	B	OR (95% CI)	P value
Age	0.015	1.015 (0.968–1.064)	0.531
Spherical equivalent	0.082	1.086 (0.98–1.18)	0.059
Axial length	0.084	0.919 (0.579–1.459)	0.721
BCVA LogMAR	-1.501	0.223 (0.069–0.719)	0.012
Pseudophakia	0.1	1.105 (0.400–3.058)	0.817
Neovascular membrane	-0.437	0.646 (0.177–2.364)	0.509
Vitreoretinal interphase alteration	0.709	2.031 (0.597–6.912)	0.257
Posterior staphyloma	2.035	7.650 (2.225–25.947)	0.001
Basal MTM stage ≥3	-1.25	0.286 (0.061–1.341)	0.112

## Discussion

MTM primarily occurs in populations with high myopia and is estimated to affect 9-34% of eyes with posterior staphyloma [11,12]. Although it was initially believed that the conditions included in MTM were very stable and benign disorders associated with mild symptoms, subsequent studies have shown that this condition can be progressive and lead to vision loss [10,13]. Therefore, in this study, we described the characteristics of MTM in a Mexican population and identified factors associated with its progression.

The demographic characteristics of patients in this study were consistent with those of a previous study, with a ratio of women to men of 4:1 and an age of >60 years on average [14]. This aligns with the results of Panozzo et al., who reported a 3:1 ratio of affected women to men and a mean age of 60 years at diagnosis [15]. Similarly, Fang et al. reported a mean age of 58 years and a female: male affectation ratio of 3:1 [6]. Therefore, our patients with MTM had a demographic profile comparable to those reported in the literature (Table 5).

**Table 5:** Studies reporting characteristics of patients with MTM. MTM: myopic traction maculopathy, no.: number, AL: axial length, SE: spherical equivalent, NA: not applicable, D: diopters

Study	No. of eyes	Male:Female Ratio	AL	SE	Progression	Regression
			(mm)	(D)	(%)	(%)
Panozzo et al.^15^	125	3:01	29.75±2.12	-16.93±5.74	NA	NA
Shimada et al.^16^	168	2:01	29.6±1.5	-14.5±3.6	11.6	3.9
Li et al.^21^	113	1.9:1	29.3 ± 1.83	-12.6 ± 4.8	43.4	16.8

The ocular profile of our patients, including SE, AL, and BCVA values, aligns with what is reported in the literature. Panozzo et al. [15] reported a mean SE of -16.93 ± 5.74 D, whereas it was -16.8 ± 6.4 D in this study. Similarly, AL in this previous study was 29.75 ± 2.12 mm, compared to 29.6 ± 2.1 mm in this study. Shimada et al. [16] reported similar findings (i.e., AL of 29.6 ± 1.5 mm and SE of -14.5 ± 3.6 D). Therefore, the ocular biometry parameters of our patients are consistent with those reported in the literature.

The average number of follow-up medical consultations (four) over two years of follow-up indicates that the patients were followed as recommended, that is, with monitoring every six months [13,17].

Our results indicate that the disease is not stable or benign. Instead, it tends to worsen and progress over time, with 25% of patients experiencing progression of the disease. According to the patient-year progression rate, 20% of patients progressed each year on average, which is similar to the findings of Kong et al., who reported an annual progression rate of 24.59% [7]. The regression rate is very low, consistent with previous observations [8]. Additionally, previous reports have indicated that surgery may not stop disease progression in some cases [18-20].

We found that a better basal BCVA (lower the LogMAR) was associated with a lesser probability of progression, but the presence of posterior staphyloma was related to a seven times increasing probability of progression. These findings are consistent with those of Li et al., who found that the initial VA predicts progression [21]. On the other hand, previous reports also found that posterior staphyloma is a highly influential factor in increasing the probability of progression [19,20]. The extent of macular retinoschisis is another factor associated with progression; however, this parameter was not measured in our patients [16].

It is important to note that our study had some limitations. Because of its retrospective nature, the follow-up intervals could not be established. Furthermore, AL could not be obtained for some patients. Additionally, the sample size was relatively small; accordingly, the results cannot be generalized.

Although East Asia is the region most affected by degenerative myopia, the problem is global, including Latin America. Therefore, this study is of utmost importance as it provides data for the behavior of degenerative myopia and MTM in the Mexican population. Future studies with larger sample sizes could provide a better understanding of the behavior of MTM in this population. Additionally, it is crucial to determine the prevalence of myopia and its complications in the Latinx population.

## Conclusions

In conclusion, we characterized the features of 101 eyes of Mexican patients with MTM and identified posterior staphyloma and BCVA as crucial prognostic factors for disease progression. The results obtained are consistent with findings reported in other populations with a higher prevalence of the disease. These findings hold significance for prognosis and surgical decision-making in individuals afflicted with MTM.
